# Platform-mediated racialization: A case study of rural Chinese wig sellers and Black clients on Alibaba

**DOI:** 10.1177/14614448251358351

**Published:** 2025-09-23

**Authors:** Shichang Duan, Shanshan Lan

**Affiliations:** University of Amsterdam, The Netherlands; University of Amsterdam, The Netherlands

**Keywords:** Alibaba, Blackness, e-commerce, platform, racialization, wig supply chain

## Abstract

Due to the rise of global e-commerce platforms like Alibaba, online interactions between rural Chinese wig sellers and Black clients are increasing. However, the racial implications of such platform-mediated business relations remain underexplored. Based on ethnographic fieldwork in Xuchang, a leading distribution and export center for wigs products in the global supply chain, this research examines the active role of Alibaba in orchestrating, structuring, and shaping the racial knowledge formation of rural Chinese wig sellers in their daily online interactions with Black clients. We argue that the exploitative nature of platform capitalism creates stress and exhaustion among rural wig sellers and pushes them to draw narrow and hasty conclusions about Blackness that dovetail with racialized hierarchy in the global wig industry and logistics networks. This research bridges the gap between platform studies and critical race studies and contributes to the reconceptualization of relations between platform and race.

This article examines how platforms such as Alibaba mediate the racialized interactions between rural Chinese wig sellers and their Black clients,^1^ focusing on how the sellers categorize their clients and construct a hierarchy among different groups of Black customers via platform metrics during online business negotiations. We focus on Alibaba because it is one of the world’s biggest e-commerce and retail platform and has played a key role in promoting rural e-commerce in China (Wang, 2024). Alibaba also developed sophisticated internationalization strategies, which connect small and medium-sized enterprises (SME) with the global market (W. Li and C. Li, 2022). The platform became even more popular for clients outside China who wish to buy affordable wig products during the Covid-19 pandemic, when international travel was suspended or strictly controlled.^2^ The data for this research was collected via ethnographic fieldwork in a Chinese wig factory in a rural area near Xuchang, a prefecture-level city in central Henan province, from November 2023 to February 2024, shortly after the end of China’s official Covid control policy. This time frame is important because it captures a moment when cross-border mobilities were still difficult and platforms such as Alibaba played a key role in facilitating international trade activities. The period from November to February also covers peak sales time such as Christmas and New Year, which is the busiest yet also the most rewarding season for our wig seller participants.

While there are a wide range of commodities involved in grassroots China-Africa trade, we focus on wig products due to their association with racialized meanings of Blackness and identity performance in both the global wig supply chain and in daily life cross-cultural encounters (Lukate and Foster, 2023; Tarlo, 2019). We pick Xuchang as field site due to its reputation as a leading distribution and export center for wigs, commanding over 60% of the global wig market (Wang and Xu, 2023). Already decades before the advent of online platforms like Alibaba, Xuchang had an advanced global wig business with a history that dates back to the Ming dynasty when a German came to collect hair and process preliminary hair products for the European market (Liu and Zhai, 2023). Today, it has more than 4000 big and small factories and 300,000 residents working in various niches and selling products to more than 150 countries and regions (Xuchang Government, 2022). Compared to other wig production sites in China, such as Qingdao and Guangzhou, wig factories in Xuchang target primarily Black customers from different parts of the world. Its niche market for Black clients is much bigger than other hair products (Tarlo, 2016).

Existing literature on African traders in China is restricted to the African markets in coastal cities such as Guangzhou and Yiwu (Bodomo, 2012; Cissé, 2015; Lan, 2017; Mathews, 2017). This research expands the scope of existing literature by focusing on a relatively poor and underdeveloped area in North China and on racialized e-commerce transactions between rural wig sellers and their Black clients. Current research on the racialization of Blackness in China’s cyber space mainly focuses on elite perspectives and its strong sense of nationalism (Cheng, 2011; Frazier and Zhang, 2014). These studies are restricted to content analysis and thus pay little attention to the mediating role of online platforms. While an emerging anthropological scholarship examines grassroots racialization in daily life interactions between Chinese and Black Africans (Lan, 2019; Sautman and Yan, 2016), online business interactions between grassroots Chinese and Blacks remain less explored. This article bridges theories on platform labor studies and South-South racialization. Instead of assuming the platform as a neutral instrument, it highlights how platform-mediated racialization facilitates the production of racial knowledge through its visibility regime.

Racialization is defined as “the extension of racial meaning to a previously racially unclassified relationship, social practice, or group” (Omi and Winant, 2014). As Monson and Rupp (2013) have shown, examining racialization not only helps elaborate the construction of racial identities but also highlights this process as contextual, situational, contingent, and inherently unstable. Existing literature on racialization in platform society mainly regards platforms as instruments or largely as backgrounds (Gebrial, 2022; McMillan Cottom, 2020; Matamoros-Fernández, 2017; van Dijck et al., 2018). For example, many scholars analyze the discursive construction and performance of race, including images and narratives across social media platforms (Banks et al., 2024; Riccio et al., 2024; Zhou, 2024). They argue that platforms amplify and reproduce, implicitly or explicitly, existing racial inequalities (Daniels, 2013; Melamed, 2015; Moran and Gatwiri, 2022). The Chinese context differs from the multiracial diversity in many Western countries due to the relative absence of racial minorities in its platform labor force. Yet the popularity of Chinese wig products among Black clients facilitates the rise of e-commerce as a new site of interracial encounters. Our case study enriches existing literature by examining the active role of Alibaba in orchestrating, structuring, and shaping the racial knowledge formation of rural Chinese wig sellers in their daily online interactions with Black clients.

The wig sellers in this research were born and raised in rural areas near Xuchang. Their age ranges from 20-30. A few of them have a bachelor’s degree, but most graduated from three-year junior colleges. These rural youth consider e-commerce a promising job with good financial returns and thus have invested much energy in learning how to work on e-platforms like Alibaba and how to interact with international clients. We propose that through marketing wig products on Alibaba and cultivating good relations with Black clients, these rural youth also engage in racial learning. We define racial learning as “the development of knowledge about racial differences and racial hierarchies through daily life experiences in various transnational, local, institutional, and community settings” (Lan, 2012: 5–6). Before their entry into the e-business, rural Chinese wig traders had little face-to-face encounters with Black clients. Their knowledge about Blacks came mainly from secondhand sources such as state media, the Internet, popular videos on Chinese social media, rumors and hearsay. The rise of e-platforms like Alibaba provides rural youth an opportunity to engage in daily communication with Black clients, and thus has the potential to facilitate more nuanced racial knowledge. This differs from the Western world where racial inequality has a notorious history that platform capitalism taps into (Benjamin, 2019; Cheney-Lippold, 2017; van Doorn, 2017). In this case, we argue that the exploitative nature of platform capitalism creates considerable stress and exhaustion among rural platform workers and pushes them to draw narrow and hasty conclusions about Blackness that dovetail with racialized hierarchy in the global wig industry and logistics networks.

Below we provide background information on the evolution of Alibaba and its role in promoting the wig industry in Xuchang. Then we discuss “platform-mediated racialization,” the theoretical lens that draws from platform and critical race studies. Next we explain our methodology. We elaborate our findings in three aspects: (1) Alibaba’s visibility regime and its impact on Chinese wig sellers’ perception of the presumed Black Other; (2) how our Chinese informants rank Black clients by creatively combining and interpreting categories provided by Alibaba; (3) how e-platforms both constrain and open up new spaces for racial knowledge formation among rural Chinese who are passionate about succeeding in the global wig industry.

## Background information

Alibaba corporate was founded in 1999 as a B2B e-commerce platform with the aspiration of helping SMEs in China directly export their products to global resellers. Various factors contribute to the growth of this tech giant, including the support of global venture capital, the state’s economy policy and ambition, and especially the existing industrial base. Zhang (2020) divides the development of Alibaba into two sages. During the first stage (1999-2007), the platform adopted a democratic and participatory model, focusing on increasing participation of petty entrepreneurs and market expansion. During the second stage (2008-2017), Alibaba’s business model became more profit-driven and exploitative through mechanisms such as datafication, selection and commodification. In 2009 Alibaba launched its “Taobao Village” initiative, with the goal of expanding its rural market. Alibaba invested a lot to facilitate international trade between rural e-entrepreneurs and global clients. For example, it developed embed instant translator in its interface, which recognizes more than 220 languages. In Xuchang, there is an Alibaba branch focusing on convincing and assisting wig factories there to join the platform. The Alibaba staff frequently attend the workshops, parties and events of these factories.

Before the rise of e-platforms like Alibaba, Xuchang was positioned at the lower end of the global wig supply chain, providing wig products to brokers in Hong Kong, Japan, and South Korea, who monopolized the sales of wig products to international clients. This unequal division of labor hindered the development of local brands and creative capacity. Until recently, most Chinese wig vendors in rural areas had no direct contact with their foreign clients. Today, Alibaba allows wig sellers in Xuchang to access foreign clients directly, to engage in cross-cultural communications with the support of translation software. Consequently, Xuchang’s position in the global wig supply chain has changed from a low-end supplier to a global wig distribution and export center. Before the world economic crisis in 2008, Xuchang’s wig products were mainly exported to Western countries. However, with the decline of orders from the West after 2008, factories in Xuchang turned to Africa as a major market for their wig products (Tarlo, 2016; Wang, 2022). This is partly enabled by e-platforms such as Alibaba, partly by state policy to boost trade relations between China and Africa.

Since the opening of the first tri-annual Forum on China-Africa Cooperation (FOCAC) in Beijing in 2000, international trade between the two parties has been rising steadily. In 2009, China surpassed the United States to become Africa’s largest trading partner (Dews, 2014). Wang (2022) notes that compared to wig markets in more developed coastal areas such as Guangzhou and Yiwu, the wig industry in Xuchang is dominated by township and village enterprises (TVEs) and involves less interprovincial labor migration and foreign direct investment. It is also less accessible for African traders due to Xuchang’s location in a remote and underdeveloped area in North China and the lack of English proficiency of rural entrepreneurs. All these factors contribute to the booming e-commerce in Xuchang, where a new generation of college-educated, technology-savvy rural youth are recruited to work in the platform economy. This younger generation often reject the labor-intensive work on the factory floor and invest a lot in the neoliberal dream of digital entrepreneurship, which celebrates individual creativity yet also promotes cutthroat competition (Zhang, 2021). This younger generation of digital entrepreneurs also contribute to the shift of Xuchang’s wig industry from OEM (original equipment manufacturer) to ODM (original design manufacturer). As observed by Wang (2022: 399), “Xuchang-made wig products for the African markets are not by-products of the supply chain serving the West but are tailored for the niches of the African markets.”

Alibaba connects Chinese wig sellers with Black clients in Africa, as well as in Europe, America, and other parts of the world. Because it is a B2B platform, these Black clients are usually resellers in their local markets.^3^ They are careful to check the credibility and authenticity of the merchants and will have several video calls before closing a deal. Chinese wig sellers must work hard to build trust and credibility with Black clients in order to secure business success. Meanwhile, they are also subjected to the rules and regulations of the platform, which monitors their performance via a complicated system of metrics and data values and in the process creates a specific type of precarious digital labor (Zhang, 2021). In the context of labor, interactions with Black clients become an important and ongoing daily routine for Chinese wig sellers and constitute a primary source of racial knowledge formation. During the Covid-19 pandemic, many factories in Xuchang were forced to shut down. However, several large factories, including Xiwang, leveraged their connections with local governments and supply networks in India, North Korea, and Bengal to continue production. These factories secured more orders during Covid and maintained a dominant position in the industry afterward.

## Platform-mediated racialization

In a study on grassroots racialization between rural Chinese migrant workers and African traders in Guangzhou, Lan (2019) distinguishes between “presumptive racialization,” which is based on secondhand racial learning, and “interactive racialization,” which is based on daily life cross-cultural interactions. This research treats the online business transactions between rural Chinese wig sellers and Black clients as racialized and embodied practices that blur the boundary between presumptive racialization and interactive racialization. It moves beyond the binary between sellers and buyers and interrogates the mediating power of the e-platform (van Schie, 2022) and its visibility regime. We propose that understanding the unequal power relations between the e-platform and the rural Chinese wig sellers is the key to grasping their racialized interactions with Black clients, which we conceptualize as “platform-mediated racialization.” We use the term to critically examine the entangled relationship between visibility politics in the platform economy, exploitative labor relations, and process of racialization. Tarlo (2019) notes a process of racialization in the production of wigs on the factory floor in Xuchang. Our research shifts the focus from the production stage to the sales stage and treats the transnational field of e-commerce as a new site of racialization.

As visibility has gradually become a source of power in platform society, Bucher (2016) reconfigures Foucault’s notion of surveillance as a form of permanent visibility to argue that platforms construct the threats of invisibility to achieve its algorithmic power, as every user should struggle for high visibility by optimizing their performance. Working harder to catch up with the evolution of platforms’ visibility rules is a common strategy, if not a routine, for digital workers desiring more branding (Arriagada and Bishop, 2021). Chinese wig dealers represent a similar labor force in the platform economy disciplined by platform metrics. Interaction with and sales to Black clients are facilitated by Alibaba featuring the wig dealers prominently to international consumers when they search the platform. The visibility of wig traders is also constantly adjusted according to metrics, such as customer rating, timely response, and sales, all of which Alibaba designs and monitors. Sellers only get their visibility increased when their interactions with Black clients generate valuable data for Alibaba, which perfectly embodies the datafied logic and profit-driven goal of platform capitalism (Poell et al., 2021; Srnicek, 2017).

Existing literature on labor relations on Alibaba has noted the highly exploitative nature of its profit-driven business model (Zhang, 2020, 2021). This research adds a racial dimension to the analysis by making a connection between Alibaba’s platform policy, affordance, algorithm metrics and rural Chinese wig sellers’ racial learning. We find that the visibility regime of Alibaba is so stressful and repressive that it has greatly constrained and condensed the length, depth, and scope of interactions between rural wig sellers and various groups of Black clients. In other words, the racial learning of Chinese wig sellers is oftentimes subordinate to the ultimate goal of increasing visibility, enhancing workplace efficiency, and reducing workload. Munn (2022) argues that information infrastructure such as data centers and undersea cables function as epistemic infrastructures that privilege some forms of knowledge while ignoring others. To a certain extent, Alibaba also functions as an epistemic infrastructure that conditions rural Chinese wig sellers’ development of racial knowledge about Black clients. It does this in two ways: first by depleting the wig sellers’ energy and forcing them to rely on secondhand knowledge about Blacks learned from popular media, textbooks, and state propaganda; second by feeding rural wig sellers with platform designed categories based on nationality and racialized world geography.

Recent scholarship examines how platform workers generate critical awareness, knowledge, and resistance tactics toward platform metrics and information asymmetries such as producing algorithm gossip (Bishop, 2019), reflecting the tiered governance structure (Caplan and Gillespie, 2020), and interpreting the visibility requirements as a game (Cotter, 2019). They take various measures to appropriate and mitigate the governance of platform architecture (Gerlitz and Helmond, 2013) and the intense working pressure (Vasudevan and Chan, 2022). Chinese wig sellers are also not passive victims of Alibaba’s visibility regime. In their limited spare time, some rural youth try to surf the Internet and browse the social media pages of some of their Black clients to learn more about their cultures and personal preferences. Although such personal friendship cultivated outside the workplace has instrumental motivations, it does open up a space for more nuanced racial knowledge formation, which is ironically facilitated by the profit-driven model of platform capitalism.

## Methodology

The data draws on 4 months of fieldwork by the first author in a Chinese wig factory Xiwang (pseudonym) in Xuchang, Henan province. Xiwang factory produces diverse hair products, such as bundles, frontals/closures, and wigs. This factory is one of the most lucrative companies in Xuchang, working on global hair product transactions for over 20 years, and owning several e-commerce teams focused on various platforms such as Alibaba, Amazon, Shein, and TikTok, respectively. Alibaba is the most important platform for this company so far, and a very popular platform among Chinese working on global transactions since 1999. Xiwang factory has over 40 wig sellers on its Alibaba team and paid over 200,000 euros annually to improve its visibility on Alibaba, which makes it a good example to investigate the wig industry in Xuchang.

The first author worked as a volunteer on the Alibaba team of Xiwang factory. He received training and was tested three times for his basic knowledge about wig products before joining the team. All members on the Alibaba team were aware of his researcher status. Data collection during the early stage of the research was mainly based on participant observation. During the second stage, twenty semi-structured interviews were conducted with wig sellers who have regular contact with Black clients. In addition, the first author also conducted thirteen interviews with various groups of Chinese working in the wig industry in Xuchang: two hair designers who are responsible for developing new style wigs, two workers in a wig factory who are responsible for curling and trimming hair, three human resource personnel, four mid- and top-level managers, and two entrepreneurs knowledgeable about e-platform rules and regulations. These interviews helped provide important background information for this research. The first author also helped Xiwang factory receive six Black clients (from the United Kingdom, Ireland, Congo, Nigeria [two clients], and South Africa) who visited Xuchang to find business partners.

Below we present our findings in three aspects. First, we explain how the exploitative nature of platform metrics creates stress, exhaustion, and fatigue among our rural participants and leaves them little time for active racial learning. Then we examine how rural wig sellers combine racialized categories provided by the platform with secondhand racial knowledge accumulated in daily life to achieve the goal of reducing workload, improving work efficiency, and enhancing visibility. Finally, we discuss self-initiated friendship between some wig sellers and Black clients, which may promise, in a limited scope, more nuanced racial knowledge formation.

## Platform metrics, work-related stress and exhaustion

**Figure 1.**
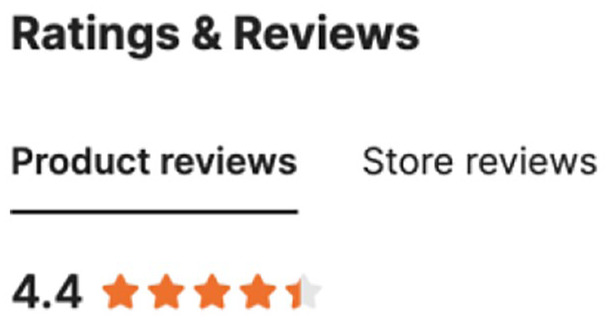
Rating metrics (screenshot from Alibaba app.).

After becoming a seller on Alibaba, the first lesson rural Chinese youth must learn is the key role of metrics on the platform. According to these informants, three types of metrics should be valued. The first is rating and disputes rates. Five stars means great service (Figure 1), which helps increase visibility. Sellers with good ratings often share their screenshots in the company’s WeChat group. This aims not only to encourage co-workers, but to stimulate competition. To some extent, high ratings from clients is more important than one-time sales because it renders both traders and products more “algorithmically recognizable” (Gillespie, 2016). The opposite of great ratings is disputed rates. Disputes usually mean refund, returning products and decreasing visibility—disastrous for sellers. The return of products in global e-commerce involves tax, long duration, and high-cost delivery. The company trained Chinese respondents to interpret negative feedback from clients as proof of one’s bad service and incapability in handling business on e-platforms. Wang, a 35-year-old mother of two, explained: “one has to be very careful and avoid potential disputes with clients, otherwise the platform would decrease your data traffic.” “Decrease data traffic” means that the platform has the power to make vendors less visible or invisible by lowering their ranking. These decisions are generally impossible to appeal. More experienced wig traders usually advise newcomers that the rating metrics, which measure the overall quality of interactions with clients, should be their number one priority.

The second significant metric is the “On-time response rate” (ORR), which calculates how many times the Chinese sellers reply to clients in five minutes. A high ORR means a great service experience, which helps Alibaba attract more consumers. ORR is important because it is directly related to the wig factory’s financial investment. It is common practice for a wig factory to pay a deposit 1 month in advance to Alibaba so that the platform can make its products more visible to potential clients. The average price for being seen by a random client is around three euros in Xiwang factory and the price for a client who shopped five times in the past 2 months will be eight euros. This monetization of ORR has created huge pressure among platform workers. The first author personally witnessed a team leader yelling angrily to an intern. He said, “You don’t know how much we paid for every client! Take it seriously, okay?” It turned out that this intern forgot to reply to a client’s message at 3 a.m.

**Figure 2.**
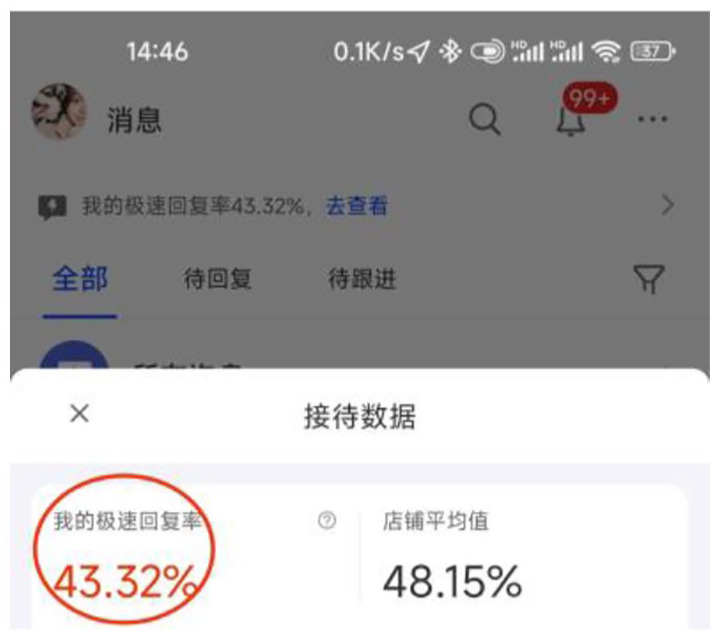
On-time response rate (43.32% is the rate of a certain agent, 48.15% is the average rate of other agents in same digital shop) (Screenshot from Hao’s mobile phone).

Many Chinese wig dealers understand why ORR is a key index to distribute visibility on Alibaba. However, they also note the challenges of improving ORR since clients are from all over the world in different time zones and their messages may emerge any time. To avoid missing messages, agents usually turn the phone volume to maximum and reply immediately to notification rings. This stringent ORR regime often blurs boundaries between work and free time and exhausts vendors. Hao, a 28-year-old father of a newborn, told me, “I must reply every night during and after caring for my son! Sometimes I lie in bed replying to clients, and my phone was the only light in the darkness.” This intense and endless labor increases the possibility of making sales for themselves, but also creates value for platforms, which is a common strategy of platform capitalism (Figure 2).

**Figure 3.**
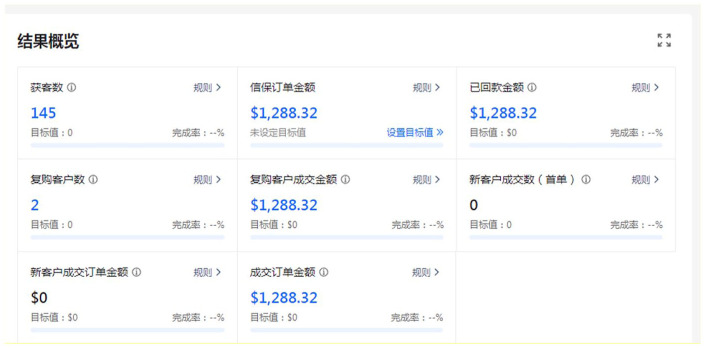
Screenshot of Yi’s sales record from Alibaba.

The third metric is the sales record of platform workers provided by Alibaba, which rewards individual vendors with more visibility for a good sales record. In many wig factories in Xuchang, a seller’s commission fluctuates with their sales record. For example, if a trader brings in 1000–10,000 dollars revenue per month, they could earn a 2% commission. The percentage will be around 5% if sales reach 10,000–30,000 dollars. While the platform workers’ basic income is only 300 euros per month, their sales record may create a huge variability in their monthly income. In Xiwang factory, more experienced agents could earn 9000 euros per month while new sellers might only glean 645 euros. Sales competition is often framed in the language of warfare. As En, a 42-year-old man who sold phones in Africa for 8 years before switching to the wig industry in 2019 put it, “why do you complain? Everybody has the same opportunity, and you must work like a solider. Nobody comes there for a basic salary and your fate is in your hands.” To a large extent, the sales record works like a number game, and gamification metrics (Vasudevan and Chan, 2022) constantly produce competition, stress, exhaustion, and frustration (Figure 3).

At first glance, these metrics (especially types 1 and 2) seem to prioritize the needs of clients and have the potential of facilitating more positive business interactions between Chinese wig vendors and Black clients. However, the time constraints and work pressure imposed on wig sellers ended up hindering more meaningful or friendly interactions. We propose that platform-generated metrics not only intensify the exploitation of rural Chinese wig sellers as platform labor, but structure and condition their development of racial knowledge concerning Black clients. Our findings show that platform-mediated-racialization involves three types of interactions between rural wig-sellers and clients: indifference, hasty categorization, and self-initiated friendship. It must be noted that these divisions are analytical in nature and they are often intertwined and cannot be easily separated.

Indifference is usually the first reaction when rural Chinese are inquired of their impression of Black clients. During interview, the first author often got answers like this, “I don’t know much about them. Mostly we only talk about business, hair products and no time to learn more.” Yi, a 19-year-old female college student, opined, “I lost my passion to learn more about foreigners shortly after I started my job. The daily chatting is interesting but useless! I must survive and have sales first, right?” Yi’s testimony reflects the predominance of the visibility regime in shaping the racial learning of rural wig sellers. In other words, interesting conversations with Black clients, which may lead to more nuanced racial knowledge are considered useless unless it serves the ultimate goal of increasing one’s sales record. We interpret such indifference not as signs of ignorance, but as symptomatic of exhaustion and fatigue due to work pressure, which discourages our participants to engage in more active racial learning. Our data shows that Alibaba’s visibility regime may lead to impersonalized, oversimplified, and fast-paced exchanges between rural sellers and clients. Since the majority of their clients are Black and the chances of encountering white clients are few, many local wig sellers use “Black” and “foreign” interchangeably in daily conversations.^4^ This conflation of Black and foreigner identities diverges from the general trend in urban China, where white Westerners are popularly perceived as the default foreigners (Farrer, 2019). The normalization of Blackness in Xuchang’s online wig business reflects the situated nature of racial formation on e-platforms such as Alibaba. It also underscores the unmarkedness of Blackness as a racialized identity in the evolving knowledge system of rural wig sellers.

## Categorizing different groups of Black clients

On December 20, 2023, during the peak season for Christmas sales, the first author attended a workshop organized by Xiwang factory. The goal was to teach platform workers how to interact with Black clients on Alibaba. The teacher was a 29-year-old woman from Zhengzhou who had worked for 6 years as an entrepreneur focusing on learning Alibaba algorithms. She started the training by empathizing with the workers for their high level of stress at work. She said, “as a human being, our energy is really limited, and we must be smart and know how to rank clients based on several categories. Otherwise, you will die because of the endless work.” She gave an example on how she handled different Black clients. First, she checked their IP address and digital traces to discover their location and how often they had shopped on Alibaba in the past 3 months. A client from the United States who shopped a lot in the last 3 months was considered a “high-quality client” and “deserved” to be carefully served. If the message was from a trader in Africa who registered days ago, she would tell the client that it was midnight in China, and she needed sleep and she would reply in 9 hours. In this way, she could focus her energy on the US client. “Remember,” She emphasized, “US clients are our priority. They are rich so they can afford expensive products. They are stupid because they are used to horrible e-commerce platforms such as Amazon with bad products and slow delivery service.” Another trainer added, “as for Africans, it’s challenging to trade with them for many reasons, so don’t count on them too much.”

This training constitutes an important moment of racial learning for rural wig sellers since it teaches them how the IP address and digital traces of clients provided by Alibaba can be reinterpreted to categorize different groups of Black clients. Since Alibaba offers the national IP address of clients, such as United States, French, United Kingdom, Jamaica, and so on, Chinese sellers pick up these categories easily and use them to differentiate clients from different parts of the world. However, the categories used by the trainers in the workshop are not based strictly on nationality, but reinterpretations that are based on overgeneralization, homogenization, and hierarchical ranking of clients according to a racialized world geography. For rural wig sellers who are positioned at the margin of the Chinese social system and who had little overseas study or travel experiences, such terms as “US clients,” “European clients,” and “African clients” are not neutral terms. They take on racialized connotations as our participants mix such national/regional categories with their secondhand knowledge about Black people, white people, foreigners, and Westerners. Overall, our findings show a hierarchical ranking of Black clients, with US clients at the top, European clients at the bottom, and African clients somewhere in the middle.

### Easy US clients versus difficult African clients

As demonstrate by the training session, rural Chinese wig merchants generally believe the stereotype that Black clients from the United States are rich and easy to deal with. This is often backed up by the fact that delivery service to the United States is fast due to the existing advanced logistical infrastructure there. The trainer’s assumption that US clients are “stupid” is based on their experience that relatively lower number of complaints comes from US clients compared to the “picky” European clients. In contrast to the “easy” US clients, Africans are constructed as difficult because they often bargain and after learning the delivery cost often cancel their purchase. African clients are usually shocked by the high delivery cost and long delivery time, mainly due to the relatively underdeveloped logistical infrastructure on the continent and corruption in the customs clearance, according to Chinese wig sellers. A common belief among Chinese sellers is that even if African clients buy wigs, they leave bad ratings and even disputes because of the horrible delivery experience. Since dealing with African clients usually takes more time and energy and does not always lead to a successful business deal, most vendors follow the trainer’ advice to prioritize US clients. Ying, a female agent earning around 30,000 dollars in sales per month after working in Xiwang factory for 3 years, remarked, “you must smartly invest and distribute your limited energy. It is easier when you have a preferred client group. Yes, I’m talking about the US clients.”

Although Ying’s decision to prioritize US clients is a strategy to reduce workload and manage the visibility politics on Alibaba, it can also perpetuate racialized bias against clients from Africa. For instance, many Chinese sellers focus on working in the morning, from 9 a.m. to 11 a.m., and from 11 p.m. to 3 a.m., popular times for US consumers to search for hair products online. Xiwang factory even set this period as their normal work time for months and only changed back under pressure from workers’ families. When a US client sends messages, Chinese wig sellers respond swiftly and recommend high-quality products. They sometimes even request US clients’ WhatsApp numbers to deepen connections. African clients are considered less important. If US and African clients send messages at the same time, the Chinese agent either ignores the African client or replies briefly. If they ask for recommendations, sellers will offer some low-end and cheap products. Chinese may ask for African clients’ WhatsApp numbers, but only “so they won’t message me on Alibaba,” explained a trader named Liu. “I can maintain a high on time response rate without replying to them. As for US clients, I contact them on WhatsApp hoping to turn them into my regular clients.”

One may argue that the differential treatment of US clients and African clients is largely determined by disciplinary platform metrics such as ORR, sales records, and good ratings. However, we want to move beyond the economic analysis by pinpointing the racial implications of such platform-mediated interactions. The binary opposition between “US clients” and “African clients” is problematic because the first category is based on nationality and the second on a region. Black clients from Africa are lumped together as a homogenized category with no distinction of nationality, citizenship, class, and gender. The complaints of African clients’ bargaining culture resonates with the racialized perception that Black Africans are poor and cannot afford high-end hair products (Mathews, 2017). To a certain extent, the claim of US clients being stupid risks perpetuating the stereotype that Blacks have lower IQs. Meanwhile the assumption that all Black clients from the United States are rich and deserve better service also reflects the privileging of nationality, citizenship, economic status, and logistical infrastructure (rather than skin color) in the racialization of US Blackness. Lukate (2023) finds that Black women from the United Kingdom who return to African countries such as Nigeria, Ghana, Uganda and Tanzania to visit are often racialized as “White.” Chinese wig sellers did not explicitly address US clients as “White,” yet their prioritization of US clients over African clients reflects their hierarchical ranking of US blackness as being structurally positioned closer to hegemonic white power.

### “Worst” European clients

Compared to US and African clients, European clients are ranked the lowest by Chinese wig dealers because they are perceived to be fussy and captious. Among Europeans clients, those from the United Kingdom and France received the most criticism from our participants. Not surprisingly, this distaste for UK and French clients is mainly based on their potential to jeopardize Chinese sellers’ visibility on Alibaba and to negatively impact their platform metrics. Zu, a 31-year-old female trader who is passionate about the wig business, said, “UK clients always leave bad comments, or ask for extra discounts for the future. The coupons are fine, but the comments are quite annoying!” Ping, another experienced female trader said, “I quite resent clients from France and even don’t want to do business with them. They always start disputes!” Sellers deploy various strategies to avoid doing business with European clients, such as increasing the price, claiming the products the clients want are out of stock, or recommending other vendors on Alibaba. Although such strategies are devised with the goal of protecting the visibility of the sellers, they can easily trigger more informal complaints.

Dealing or not dealing with European clients became a catch 22 for rural Chinese platform workers, who may sometimes resort to highly derogatory and racialized languages to describe clients from the United Kingdom and France. For instance, Lie, a 25-year-old male wig seller, showed strong resentment toward UK clients. He said “I hate them. They are just like their white masters and think they can lord over us! They believe they deserve the best price, service, and quality.” Lei’s intense feelings need to be contextualized by the highly repressive visibility regime of Alibaba. Faced with enormous pressure for better platform metrics, Lei was most likely venting his frustration on some “bad” clients. However, in this quote Lei also displays complicated nationalistic sentiment by evoking racialized images such as Black slaves, white masters, and China’s semi-colonial history. By accusing an imagined Black UK client of imitating the arrogant behaviors of a white colonizer, Lei endorses the racial stereotype that Blacks are inferior to whites. Yet Lei’s resentment of white arrogance toward Chinese also resonates with the rise of nationalism and xenophobia in post-Covid Chinese society.

In their research of online Chinese attitudes toward Westerners during Covid-19, Ang and Martin (2024) identify a connection between state-led patriotism, which emphasizes China’s history of “national humiliation” under Western powers, and the racialization of white foreigners. Although rural Chinese wig vendors have little chance to encounter foreigners in person, they are well educated (via history textbooks and state propaganda) about China’s history of “national humiliation” at the hands of Western powers such as the United Kingdom and France. A dealer named Ying complained to the first author about a client from Ireland, who was always suspicious, unreasonable, and demanding regarding delivery time. Unaware of the historical conflicts between Ireland and the United Kingdom, Ying collapsed the two categories into one. She said, “now I remember why our history textbook describes the UK as Western hegemony.” Rural Chinese wig traders’ resentment toward UK and French clients is also mediated their geopolitical imaginary generated from news reports. A female agent half-jokingly told the first author “many news [reports] said that there are protests in France every day. No wonder it’s such a mess—because its residents are picky!”

In this section, we examines how Chinese wig sellers mix some of the categories provided by Alibaba and its training staff with their own secondhand racial knowledge to hierarchically rank Black clients. We find that platform-mediated racialization often blurs the boundary between presumptive racialization and interactive racialization since the two can happen simultaneously due to the fast-paced work environment and the disciplinary power of platform metrics. While the racialization of African clients involves a higher degree of homogenization and overgeneralization, we also find the absence of prevailing stereotypes against Black Africans such as illegality, criminality, and hypersexuality, which have been extensively covered in the literature on Africans in Guangzhou (Castillo, 2020; Jordan et al., 2021; Lan, 2016).

## Self-initiated friendship

So far we have argued that rural Chinese wig sellers’ racialized stereotypes against Black clients are implicitly facilitated by the visibility regime of Alibaba. Here we want to propose that they are not merely passive victims of the platform metrics. Our participants clearly understand the exploitative relations and divergent interests between them and the e-platforms. Many traders reported that visibility does not necessarily bring sales and profits. They gradually realized the limitations of using platform-provided labels to categorize clients and the need to cultivate friendships and long-term relations with potential clients. Some voluntarily follow the social media profile of some clients in order to know more about their daily lives and aesthetic preferences. Some Chinese vendors also share life stories and pictures with clients, such as food, landscape, and small cute decorative objects. They also ask Black clients to share their lives, which is helpful to build connections.

Such efforts to cultivate friendship sometimes proves interesting and eye-opening. One Chinese girl mentioned a Black client from Congo, who selected a wig for his wife as a surprise gift for their wedding anniversary. During video calls this client spoke slowly and smiled frequently, which made her feel quite happy. She said, “they are just like us and some of them are even better. So lovely and nice!” This girl’s positive experience with the client forms a contrast with the indifferent and business like attitude of some of her colleagues. It shows that there is not necessarily a contradiction between building friendly relations with clients and boosting one’s sales record. Another trader always sends Christmas gifts and cards to his clients, but once he received a serious message from a Muslim client from the Middle East, telling him to stop sending gifts during Christmas time. This Chinese man told the first author that he was very surprised to see the message because he thought all Western people celebrate Christmas. Yet that experience made him realize the religious diversity among his clients.

It is difficult to assess the long-term impact of self-initiated friendship with clients on the racial learning of rural Chinese platform workers since our data only consist of a few positive cases. However, we already see the potential of building more nuanced racial knowledge in these few cases. Clients from Africa are identified by their nationality (i.e. Congo) and nice personality. Clients from the Middle East are remembered due to their Muslim faith. Some sellers came to the realization that not all African clients are poor and some are quite smart and professional. Others noted that treating some clients with contempt would not help their business to grow. We do not want to exaggerate the role of personal friendship in contributing to the demystification of racial stereotypes, yet we want to acknowledge its potential in helping develop more nuanced racial knowledge within and beyond the highly regulated visibility regime of Alibaba.

### Conclusion: the contradictory role of the platform

This article examines the interactions between rural Chinese wig vendors and their imagined Black clients on Alibaba. We argue that, far from being a neutral tool, the platform mediated the racial knowledge formation process of Chinese wig dealers through its exploitative labor regime and profit-driven business model. Our findings show that platform metrics render business transactions into a competitive game of datafication, which caused stress, exhaustion, and frustration among Chinese participants. One distinctive feature of platform-mediated racialization is the intertwinement of presumptive racialization and interactive racialization. To save time and energy, the traders often mix their presumptive racial knowledge with platform-generated categories in their handling of clients from different parts of the world. This results in simplification, racialized stereotypes, and hierarchical ranking of diverse groups of Black clients, which overlaps with the global logistical and infrastructure hierarchy based on regional inequalities in development. Compared to Black clients from the United States, United Kingdom, and France, who are identified by their nationality, clients from Africa are lumped together using one homogenized category “African clients.” Nevertheless, due to their marginalization in the Chinese social system, rural Chinese wig sellers generally regard African clients as a resource that can potentially bring business opportunities and financial return. This differs from the stigmatization of Black Africans as a threat to social order and public safety by middle class urban Chinese in Guangzhou (Lan, 2015).

Our research contributes to existing literature on platform studies and the racialization of Blackness in China in several ways. First, this research complicates the relationship between platform capitalism and race. It examines how e-platforms such as Alibaba facilitate the construction of racial knowledge, rather than just building on existing racial inequality. Second, it bridges the gap between critical race studies and platform labor studies by examining the contradictory role of the platform in grassroots racialization. On one hand, Alibaba opens up a space for rural wig sellers to have direct access to Black clients from different parts of the world. This may promise upward mobility opportunities for underprivileged rural youth and more nuanced racial knowledge formation. On the other hand, the platform also offers a highly constrained space where rural wig sellers are physically, emotionally, and financially exploited and their chances of engaging in active racial learning are severely curtailed. Finally, this research contributes to literature on South-South racialization by moving beyond the Euro-American framework and foregrounding the marginalized voices of rural Chinese wig sellers, who constitute a new generation of platform labor in the global wig supply chain. Our data confirm findings in existing literature on the normalization of Blackness in grassroots Chinese/African interactions (Lan, 2016). Yet we also complement existing research by noting the invisible work of white hegemony in structuring the racial learning of rural youth, that is, the ranking of US clients as superior and more desirable than African clients, the evoking of the colonial history of the United Kingdom and France in China.

While this article is based on a Chinese case, the notion of platform-mediated racialization may be deployed to investigate the racial implications of interaction impacted by platformization in the future. For example, while much research on the platformization of cultural production focuses on the relation between digital influencers and audiences in a homogeneous cultural background, the diverse global landscape and contrasts between the West and East, are rarely discussed. Furthermore, how marginalized social groups appropriate and utilize technology to conduct cognitive activities remain a relatively unexplored topic. It involves how platforms work as the epistemic infrastructure and shape the process of knowledge production (Munn, 2022).

## Notes

1. Although not all the online wig buyers are Black, our Chinese participants tend to racialize wig products as Black and imagine their clients as mostly Black since Xuchang’s hair products mainly target Black clients.

2. Our rural wig seller participants reported an upsurge of Black clients on Alibaba since 2020, when some wig factories were shut down due to the pandemic and Alibaba became the major trading platform for wig products from China.

3. Based on our research data, individual Black clients constitute a minority.

4. Non-Black customers are identified by their nationality or ethnicity specifically, such as British or Arab.
